# Impact of Statement-Concordant Implantable Cardioverter-Defibrillator Programming on Therapy Reduction in an Asian Cohort

**DOI:** 10.1016/j.jacasi.2025.08.009

**Published:** 2025-09-24

**Authors:** Jiro Koya, Taro Temma, Kei Kawakami, Masahiro Kawasaki, Kintaro Shimano, Shota Saito, Daishiro Tatsuta, Kotaro Nishino, Takahide Kadosaka, Taro Koya, Motoki Nakao, Masaya Watanabe, Kiwamu Kamiya, Toshiyuki Nagai, Toshihisa Anzai

**Affiliations:** a Department of Cardiovascular Medicine, Faculty of Medicine and Graduate School of Medicine, Hokkaido University, Sapporo, Japan; b Department of Cardiovascular Medicine, Hokko Memorial Hospital, Sapporo, Japan

**Keywords:** implantable cardioverter-defibrillator (ICD), ICD shock, ICD therapy, mortality, statement-concordant (SC) programming

Although implantable cardioverter-defibrillators (ICDs) reduce the risk of sudden cardiac death, unnecessary therapies remain a concern because of pain, psychological burden, and potential myocardial injury. To address this, the 2015 HRS/EHRA/APHRS/SOLAECE Expert Consensus Statement, along with its 2019 update, recommended programming strategies aimed at delaying or avoiding shocks for self-terminating arrhythmias.^1^^,^^2^ Although widely adopted in Western practice, their applicability and effectiveness in Asian populations—where nonischemic cardiomyopathy (NICM) and secondary prevention ICD use are more common^3^^,^^4^—remains unclear.

We aimed to evaluate whether expert consensus-concordant ICD programming reduces ICD therapies in a real-world Asian cohort.

This retrospective study included 355 patients who underwent an initial ICD implantation at Hokkaido University Hospital between January 2013 and July 2024. The study was approved by the institutional ethics committee (Local ID: 018-0343). Patients were classified into statement-concordant (SC) or nonstatement-concordant (NSC) groups based on adherence to the 2015/2019 HRS/EHRA/APHRS/SOLAECE expert consensus recommendations. Following 1:1 propensity score matching (PSM), 126 patients (63 per group) were included for analysis. The propensity score was calculated using a logistic regression model, with the SC vs NSC as the dependent variable and clinically relevant covariates as independent variables (age, atrial fibrillation history, amiodarone use, device type, etiology, follow-up period, sex, indication, left ventricular ejection fraction, NYHA functional class). One-to-one nearest neighbor matching without replacement used a caliper of 0.2 SD of the logit.

Before November 2021, device settings primarily followed the manufacturers’ defaults, with final adjustments made at the implanting physician’s discretion.^5^ Typically, the ventricular fibrillation zone detected heart rates >185 to 200 beats/min, with initial shock of ≥30 J. The ventricular tachycardia zone was set at 150 to 170 beats/min, with up to 3 antitachycardia pacing (ATP) sequences before shocks of 10 to 20 J. After November 2021, the SC-based programming became the institutional standard, incorporating the manufacturer-specific settings aligned with the expert consensus, including higher rate cutoffs (eg, ventricular fibrillation ≥188 beats/min), longer detection intervals (eg, 30/40 intervals), and ATP-first strategies. The device manufacturers included Boston Scientific, Medtronic, Abbott, Biotronik, and Microport. The primary endpoint was the incidence of all ICD therapies (both appropriate and inappropriate shocks or ATP). Secondary endpoints included appropriate therapies and inappropriate therapies separately. Follow-up time was calculated from the date of ICD implantation to the first occurrence of the event of interest, death, or the last follow-up date, whichever came first. Patients without events were censored at their last confirmed follow-up date. Cumulative incidence curves were estimated using Fine-Gray competing risk models, treating death as a competing event. Subdistribution hazard ratios (sHRs) and 95% CIs were calculated. The proportional hazards assumption was tested using Schoenfeld residuals. All statistical analyses were performed using R version 4.4.0 (R Foundation for Statistical Computing).

Before 1:1 PSM, the median follow-up duration in the full cohort was 35.0 months (Q1-Q3: 18.0-69.0 months). After matching, 126 patients (63 per group) were included; 96 of 126 (76.2%) were men, and NICM was the predominant etiology (88 of 126, 69.8%). Follow-up period durations were comparable between the 2 groups (NSC: 26.0 months [Q1-Q3: 13-41 months], SC: 18 months [Q1-Q3: 8-33 months]; *P =* 0.272). The proportional hazards assumption was not violated *(P =* 0.45).

Overall, 28 of 126 patients (22.2%, 95% CI: 15.5%-30.1%) received ICD therapies. Those included 21 of 63 patients (33.3%; 95% CI: 22.3%-46.0%) in the NSC group and 7 of 63 (11.1%; 95% CI: 4.6%-21.6%) in the SC group. Appropriate therapies occurred in 15 of 126 patients (11.9%; 95% CI: 6.8%-18.9%): 12 of 63 (19.0%; 95% CI: 10.3%-30.9%) in the NSC group and 3 of 63 (4.8%; 95% CI: 1.0%-13.3%) in the SC group. Appropriate shocks were delivered in 15 of 126 patients (11.9%; 95% CI: 6.8%-18.9%), of whom 14 of 63 (22.2%; 95% CI: 12.9%-34.7%) were in the NSC group and only 1 of 63 (1.6%; 95% CI: 0.0%-8.4%) in the SC group. Inappropriate shocks were observed in 5 of 126 patients (4.0%; 95% CI: 1.3%-9.1%), including 2 of 63 patients (3.2%; 95% CI: 0.4%-11.0%) in the NSC group and 3 of 63 patients (4.8%; 95% CI: 1.0%-13.3%) in the SC group. During the follow-up, 13 of 126 patients (10.3%; 95% CI: 5.6%-17.0%) died, including 10 of 63 (15.9%; 95% CI: 8.0%-27.0%) in the NSC group and 3 of 63 (4.8%; 95% CI: 1.0%-13.3%) in the SC group.

The cumulative incidence of all-ICD therapies was significantly lower in the SC group than in the NSC group (sHR: 0.363; 95% CI: 0.155-0.850; Gray-test *P =* 0.014) (Figure 1A). Similarly, appropriate therapies were significantly reduced in the SC group (sHR: 0.270; 95% CI: 0.076-0.958; Gray-test *P =* 0.029) (Figure 1B). In contrast, inappropriate therapies did not differ significantly between the groups (sHR: 0.853; 95% CI: 0.192-4.087; Gray-test *P =* 0.870). There was no significant difference in all-cause death between the 2 groups (HR: 0.36; 95% CI: 0.10-0.1.30; log-rank *P =* 0.119).

**Figure 1:**
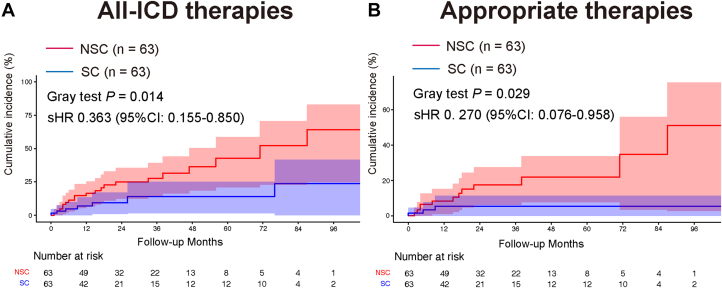
ICD Events After 1:1 Propensity Score Matching Kaplan-Meier survival curves showing the cumulative incidence of the endpoints of all implantable cardioverter-defibrillator (ICD) therapies (A) and appropriate therapies (B). NSC = nonstatement concordant; SC = statement concordant; sHR = subdistribution hazard ratio.

This study showed that ICD programming aligned with the expert consensus recommendations significantly reduced the ICD therapy burden in an Asian population. These findings were consistent with the prior studies from Western populations and provided supportive evidence for the broader adoption of expert consensus programming in Asian settings, especially given the higher prevalence of NICM.^6^, ^7^, ^8^ Delayed detection and ATP-first strategies likely contributed to avoiding the treatment of self-terminating arrhythmias, preserving the myocardial function, and reducing physiological distress. Notably, 1 case of therapy failure occurred because of ventricular tachycardia below the detection threshold after initiation of antiarrhythmic therapy, underscoring the need for individualized programming updates.

Although PSM balanced baseline characteristics, follow-up duration was shorter in the matched cohort, likely reflecting more recent treatments. Temporal bias may have existed, because SC programming was implemented more recently, potentially coinciding with the greater use of angiotensin receptor-neprilysin inhibitors and sodium-glucose cotransporter 2 inhibitors. Although the Fine-Gray model did not explicitly account for the matched structure (eg, via frailty term or stratification), propensity score matching was performed to reduce confounding. Lack of variance adjustment for matched pairs remains a limitation.

Expert consensus-based ICD programming significantly reduced both all and appropriate ICD therapies in an Asian cohort. These findings support the broader adoption of expert consensus-based strategies in diverse practice settings.

## Funding Support and Author Disclosures

This work was partly supported by the Japan Society for the Promotion of Science Grant in Aid for Scientific Research (KAKENHI) Grants (grant number JP23K15119 to Dr Temma), and the Uehara Memorial Foundation (grant number PK420950 to Dr Anzai). The authors have reported that they have no relationships relevant to the contents of this paper to disclose.
